# Risk Communication as a Tool for Training Apprentice Welders: A Study about Risk Perception and Occupational Accidents

**DOI:** 10.1100/2012/140564

**Published:** 2012-12-30

**Authors:** Marta Regina Cezar-Vaz, Clarice Alves Bonow, Laurelize Pereira Rocha, Marlise Capa Verde de Almeida, Luana de Oliveira Severo, Anelise Miritz Borges, Joana Cezar Vaz, Claudia Turik

**Affiliations:** ^1^School of Nursing, Federal University of Rio Grande, Rio Grande, RS, Brazil; ^2^Federal University of Pampa, Uruguaiana, Rio Grande, RS, Brazil; ^3^School of Chemistry and Food, Federal University of Rio Grande, 96201-900 Rio Grande, RS, Brazil; ^4^Federal Institute of Education, Science and Technology of Rio Grande, Rio Grande, RS, Brazil

## Abstract

The present study has aimed to identify the perceptions of apprentice welders about physical, chemical, biological, and physiological risk factors to which they are exposed; identify types of occupational accidents involving apprentice welders; and report the development of a socioenvironmental education intervention as a tool for risk communication for apprentice welders. A quantitative study was performed with 161 apprentice welders in Southern Brazil in 2011. Data collection was performed via structured interviews with the apprentice welders about risk perception, occupational accidents, and time experienced in welding. The data were analyzed using SPSS 19.0. The participants identified the following risk types: physical (96.9%), chemical (95%), physiological (86.3%), and biological (51.5%). In this sample, 39.7% of apprentice welders reported occupational accidents and 27.3% reported burning. The inferential analysis showed that the frequency of risk perception factors increases with the length of experience, and apprentice welders who have experienced accidents during welding activity perceive a higher amount of risk factors than those who have never experienced them. It is concluded that apprentice welders perceive risks and that they tend to relate risks with the occurrence of occupational accidents as an indicator of the dangerous nature of their activity.

## 1. Introduction

This paper discusses the perceptions of apprentice of the risks they are exposed to during the welding activity. It also presents the occurrence of accidents in this activity and the development of a socioenvironmental education intervention as a tool for risk communication (RC) for apprentice welders. In different countries, for example, Nigeria [1], Sri Lanka [2], France [3], Denmark [4], Turkey [5], and Brazil [6], the issue involving the health and safety of welders is being discussed. 

The motivation for the proposed paper came from a literature review about the theoretical approach of risk perception (RP) [7–9]. On this occasion, the researchers observed the coherence and the need to analyze the issue of human risk in different environments, among these, the apprenticeship environment, in relation to apprentices, which includes social, cultural, and political aspects in its production and reproduction [10–12].

Specifically, the interest in studying RP of apprentice welders is because the belief holds that the apprenticeship process represents a moment for dialogue, with the capacity of the dissemination of knowledge and the application of technology related to workers' health and the environment. In other words, during the apprenticeship, the apprentices should be encouraged to apply the knowledge learned about their health and on the future work environment. Besides this, it is believed that within this apprenticeship process, perceptions can be changed, from the comprehension of scientific knowledge and individual and collective behavior, which can assist workers to produce healthy work environments. Primarily, the change or the creation of awareness about health, illness, and work can be enhanced in the apprenticeship process with the aim of directing the perception of what may or may not influence or even determine an injury, an illness, or better health conditions for workers and their work environment.

The literature regarding apprentice welders shows the concern about the achievement of improving welding techniques [13–15]. Specifically, in the area of health, the investigations include genetic disorders, respiratory problems, and exposure to metals. At first, a research about chromosomal aberrations in military apprentice welders in Aberdeen, MD, USA, exposed to oxide ozone was discussed. Blood samples were collected from 273 apprentices for a period of 12 weeks. No statistically significant increases in chromosomal aberrations were found [16]. Cohort study aimed to determine the incidence of probable occupational asthma, bronchial obstruction, and hyperresponsiveness among 286 students entering an apprenticeship programme in the welding profession. The incidence of probable occupational asthma was 3%, 11.9%, of bronchial hyperresponsiveness, defined as >3.2-fold decrease in the provocative concentration, causing a 20% fall in the forced expiratory volume in one second from baseline to the end of the study. These results show that exposure to gases and welding fumes is associated with changes in respiratory function [17]. However, a study sought to identify neuropsychological effects of low levels of exposure to manganese. The cognitive performance, motor control, and psychological tests were performed and assessed for 46 apprentice welders at a local union welding school. Although the levels of manganese exposure were low, neuropsychological effects can become manifest, especially in relation to mood, attention, and fine motor control [18]. 

A search of the literature showed that there are texts that present apprentice welders as subjects, covering the welding technique and health of the subjects. The improvement of welding techniques contributes to the reduction of accidents during this activity, as it does regarding possible injuries and accidents as a result of the welding activity. However, there were no texts that show that RP is related to the activity of apprentice welders and the concern with the apprenticeship process about health and safety at work, from of theories of RP and RC because the approaches are necessary for the system management of risk to human health at work. 

According to the theoretical orientation, this research assumed that the notion of risk perception involves two factors: the magnitude of the potential loss and the probability of its occurrence [19]. In other words, the existence or not of different risk factors and occupational accidents might explain why people perceive the same risk in very different situations or why the same individual might perceive risk differently, depending on when he or she is asked about it [20]. 

 Risk perception encompasses both personal and work environment-related ideas and constructions because, to perceive it, you have to believe it [19]. Therefore, the study of apprentice welders' RP is important, as individuals are responsible for the risks perceived in the work environment. That individual might have caused the risk which an individual perceives. This creates the possibility of changes to minimize or even eliminate risk factors related to individual behavior or even in their own working conditions. One of the processes of interaction to promote the various changes may be the tool of RC.

RC is here understood as an interactive process of exchange of information and opinion among individuals, groups, and institutions [21]. RC can also help promote changes in individual and collective behavior. RC theory and practice may include public participation and conflict resolution. RC, as aforesaid, was used as a tool for the development of a socio-environmental education intervention with apprentice welders. 

Another theoretical orientation is a classification of different risk factors that the apprentice welders are exposed to. Therefore, the Act of 16 June 1999 [22] was used, which provides occupational hygiene and safety standards and the obligations of employers and employees to create a safe work environment, organization of hygiene and safety at the level of the enterprise, institution, and state, procedures for settlement of disputes in this matter and responsibility for breaches of established standards. In the specific case of apprentice welders, during welding activities, they are exposed to various occupational risks generated by chemical, physical, biological, and physiological risk factors. 

Physical risk factors to which apprentice welders are exposed include noise from welding machines and the heat of the flame from the burning of a gas mixture. Chemical risk factors include contact with different metals. Biological risk factors may be related to inflammation of the ear due to the use and exchange of earplugs. Physiological risk factors are incorrect posture during welding activity because the apprentice welders perform the activity on a flat piece of metal and they must move around the piece to hold the solder. This characteristic of the welding process causes the apprentice welders to have postures for better results of welding which are not necessarily ergonomically correct. 

These factors can create or worsen occupational diseases and accidents, which depend on the nature of the risk, the degree of exposure, a lack of protective measures, work conditions and rhythms, and the worker's function [23]. An occupational accident is defined as a fire, explosion, or another occurrence at work, which may endanger the life or health of employees or that of other persons [22]. In this study, it is understood that the welding apprenticeship constitutes a time of preparation for work, so accidents that may occur in this environment will be occupational accidents. 

The welding activity in the workplace or in the apprenticeship environment promotes the occurrence of accidents caused by different risk factors. In other places, it is possible to identify health problems related to noise, provoking irritability of the worker, physical stress, and decreased hearing ability [24], among others. The inadequate postures, the long working hours standing, and repetitive movements may cause injury and pain in the cervical spine and upper and lower limbs [25]. Contact with chemicals, metal solids, or fumes are singled out as a major concern in the apprenticeship environment. Exposure to different chemicals, such as chromium, is associated with the incidence of lung cancer [26]; cadmium is related to renal dysfunction [27]; and copper, manganese, and molybdenum are associated with respiratory disorders [28]. Chemical hazards are recognized in the literature and in different studies as a risk of greater magnitude; however, it is important to identify the apprentice welders' perceptions of exposure during welding activity in order to be able to understand all risk factors in relation to their workday apprenticeship. 

For these reasons, the present study has aimed to identify the perceptions of apprentice welders about physical, chemical, biological, and physiological risk factors to which they are exposed; identify types of occupational accidents involving apprentice welders and; report the development of a socio-environmental education intervention as a tool for risk communication to apprentice welders.

## 2. Methods

This study consists of two phases. The first phase is a quantitative, exploratory, and descriptive study involving apprentice welders, conducted in 2011 in Rio Grande (RS, Brazil). The second phase consists of the report of a socio-environmental education intervention as a tool for RC for apprentice welders enrolled in this study from the results obtained in the exploratory study (first phase), conducted in 2011 in the same region.

This study is part of a larger research project entitled “*Health, Risks and Occupational Diseases: An Integrated Study in Different Work Environments*” [29]. It was approved by the Research Ethics Committee of the Federal University of Rio Grande (Universidade Federal do Rio Grande—FURG). Apprentice welders were included in the study after signing an informed consent agreement. The study was conducted using public funds (National Counsel of Technological and Scientific Development—CNPq) and linked to the Laboratory of Socioenvironmental Process Studies and Collective Production of Health (LAMSA) research group of the Nursing School of the Federal University of Rio Grande.

### 2.1. Subjects

The study subjects were apprentices enrolled in the technical programme for training as welders in Rio Grande (RS, Brazil). Eleven classes from the technical programme were invited to participate in the study. All agreed to participate. Of the total 162 apprentices, 161 agreed to participate in the first phase of the study, representing a response rate of 99.3%. 

For the second phase, consisting of a socio-environmental education intervention as a tool for RC, six classes (86 apprentice welders) were invited, all of which participated in the first phase. In addition to the apprentice welders, six members of the research group LAMSA also participated, as mediators of the socio-environmental education intervention.

### 2.2. Questionnaire and Data Collection

The first phase of the study was conducted, based on the following questions: how apprentice welders perceived the risks to which they are exposed and which occupational accidents apprentice welders reported as incurred by them during welding activity? From the theoretical basis assumed in the study, the existence of a relationship between RP and accident involvement by apprentice welders was assumed. Data collection was performed in 2011, through a structured interview from a questionnaire, composed of mixed questions—multiple-choice and single-choice. 

The structured questionnaire had multiple-choice and single-choice questions corresponding with the following variables: participant characteristics (gender, age, skin colour/ethnic origen, level of schooling, and marital status); time of experience in welding; RP among apprentice welders (the identification of chemical, physical, biological, and physiological risk factors); the occurrence of occupational accidents self-reported by apprentices.

Upon the completion of the first phase of the research, the authors organized a Socio-environmental education intervention (SEI) in the study group (second phase). The results of the first phase were used to develop RC concerning the risk factors of the work environment as an apprenticeship tool to help apprentice welders for the promotion of individual and collective health in the workplace. After analyzing these data, the issues to be developed during SEI with apprentice welders were organized. The topics included occupational risk generated by chemical, physical, biological, and physiological factors; risk perception and occupational accidents; prevention of accidents or health problems. To work out these issues, the approach of the theme of perception of risk factors was focused upon (physical, chemical, biological, and physiological) to which apprentice welders are exposed and accidents arising from the characteristics of the welding activity performed by them. This was achieved through discussion with apprentices about personal protective devices (PPD) that could minimize the risk exposure of the apprenticeship/work environment risks and possible strategies identified by apprentices to minimize the risks.

### 2.3. Data Analysis

The Statistical Package for Social Sciences (SPSS) software version 19.0 was used to organize and analyze the data (first phase). Firstly, descriptive analysis was made and then further inferential analysis was performed. The relation between time of experience in welding and apprentice welders RP was analyzed using the Spearman correlation coefficient. To verify if the apprentices who have suffered an accident at work differ in the time they are exposed to such accidents with those apprentices who have never suffered an accident, the nonparametric Mann-Whitney test was performed. 

For organization of SEI, the results from the research (first phase) were used together with scientific research in the literature to support the targeted intervention for the risks in the welding apprenticeship environment. The scientific research was structured, based primarily on Diseases Work-Related Manual, American Welding Society, Act of 16 June 1999, documents of the World Health Organization, and the Prevention Report of RC [21–23, 30, 31]. These documents include aspects of the health surveillance of workers exposed to different risk factors in their work environment and the prevention of exposure to these factors.

For the development of SEI, the principles of RC were used as follows: confidence in the message that is being developed, (in this way the workgroup can trust the content which is being presented); reiterate the scientific evidence with examples of the daily work of the workgroup as identification of the real experience of the apprentice/worker; diversify the examples in order to show different realities with similar results, clearly, briefly, and effectively; promote useful information about the objectives of the workgroup, which is relevant to understanding the intended message; recognize that the group generally do not appreciate uncertainty expressed in numeric terms and that this may require more detailed explanation. Also be sure to use clear, nontechnical language to discuss risks and other specific information, indicating the nature, form, severity, or magnitude of the risk.

## 3. Results

### 3.1. Participant Characteristics

The sample included 161 apprentice welders enrolled on the technical programme for training as welders in Rio Grande, RS, Brazil. Most apprentices 132 (82%) were male; 95 (59%) were ethnically white; 87 (54%) were single. Their ages ranged from 18 to 56 years, with an average of 28.46 years (±7.14). 84 (52.2%) had finished secondary school (Table 1). Regarding time of experience in welding, 97 (60.2%) had none, 60 (37.3%) had experience, and 4 (2.5%) did not answer the question on experience. The average of time of experience ranged from 2 to 204 months, with a mean of 28.13 months (±38.98).

### 3.2. Risk Perception

The results of the questionnaire on RP in the welding apprenticeship environment showed that 156 (96.9%) apprentice welders identified physical risks; 153 (95%) chemical risks; 139 (86.3%) physiological risks; 83 (51.5%) biological. Among the risk factors identified, the most frequent was the heat during welding activity, cited by 128 (79.5%) apprentice welders (Table 2).

A correlation analysis was carried out between the amount of perceived risks and time experience of the apprentice, via the coefficient of Spearman (*P*). The results show that the frequency of perception of physical risk factors (*ρ* = 0.201; *P* = 0.011) and physiological (*ρ* = 0.217; *P* = 0.006) increases with the length of experience. The analysis of chemical and biological risk factors showed no significant correlation with time of experience in welding. Please note that when tested on the set of risk factors (physical, chemical, biological, and physiological), a statistically significant correlation coefficient (*ρ* = 0.256; *P* = 0.001) was also shown.

### 3.3. Occupational Accidents

Occupational accidents were reported by 64 apprentice welders (39.7%). Of these, 42 (26.1%) occurred during apprenticeship activity, 21 (13%) occurred during welding remunerated activity, and 01 (0.6%) reported the accident during welding activity at home. The most frequently reported occupational accident was skin burns during welding activity, which was reported by 44 apprentice welders (27.3%) (Table 3).

The nonparametric Mann-Whitney test showed that among apprentices with experience in welding, the group that had suffered an occupational accident had greater exposure time (median = 27 months) than the group that had not suffered any occupational accident (median = 12 months), and this difference was statistically significant (*P* = 0.012). 

Aiming to investigate whether RP is different for apprentices who have suffered an occupational accident, the data were submitted to the Mann-Whitney test. From the result (*U* = 2128.000; *P* = 0.039), it is noted that apprentices who have experienced occupational accidents perceive a higher amount of risk factors (median = 9) than those who have never experienced them (median = 7).

### 3.4. Socioenvironmental Education Intervention with Apprentice Welders

The SEI included the participation of 86 apprentice welders and six researchers from LAMSA. Six apprenticeship workshops (AW) were conducted, each one with a class of apprentices enrolled on the technical programme for training as welders in Rio Grande (RS, Brazil). The time used for the planning was 40 hours with 4 hours for holding each of the AWs, totalling 24 hours. Each class had about 14 apprentices. The AW occurred where apprentices had lectures for training as welders. Also, as mentioned before, this practice included the Health Promotion in Different Work Environments Programme (HPDWEP), of LAMSA, the School of Nursing, the Federal University of Rio Grande, RS, Brazil. The HPDWEP consists of a set of coordinated actions and continuous shaft in promoting social and environmental health in different work environments, the environments of which are included in the study group's academic LAMSA.

The SEI was developed, based on the RC concept [7–9, 32]. The content (message) about the nature of risk was developed through the classification of risk factors (physical, chemical, biological, and physiological) and occupational health and safety legislation of the Brazilian Ministry of Health, the Occupational Safety and Health Act of 16 June 1999 of the International Labour Organization (ILO).

### 3.5. PPD Used during Welding Activity

To trigger the development of communication (first step) with the apprentices who are participating in the intervention, the following question was used: what PPD is used during the welding activity? The responses were expressed on a whiteboard for viewing by all the apprentices. The answers were welding cap, welding apron, welding coat, welding boots, earplugs, welding trousers, welding goggles, welding mask, breathing mask with filter, and welding gloves. This promoted the manifestation of the apprentices to make comparisons, considerations, and suggestions on the subject. There were comparisons about the PPD used by the apprentices because some only use the welding coat and trousers (provided by technical programme for training) and others use items not included in the PPD supplied by the technical programme for training, for example, the welding apron (individual purchase), in order to increase protection. Moreover, some apprentices do not use the breathing mask with filter because it is uncomfortable, which generated discussion among the participants of the AW. 

To continue the process of RC, visualization of PPD used to perform the welding activity made it possible to show the different body systems (integumentary, respiratory, and auditory) protected by PPD. Besides these, the musculoskeletal system was included, which, despite not being protected by PPD, requires attention during welding activity. The integumentary, respiratory, musculoskeletal, and auditory systems were presented to apprentices, to weld as the anatomic-physiological characteristics, risk factors present in welding activity detrimental to the health systems and recommendations for apprentice welders.

During the presentation of the integumentary system, concerns about the physical risk factor, nonionizing radiation and chemical risk factors, due to frequent skin contact with metals were focused upon. Apprentices were asked about the composition of the wire used to perform the welding. They used the wire called E71T-1, which is composed of carbon, manganese, silicon, phosphorus, and sulfur. It was emphasized that every time that apprentices have skin contact, or by touching the metal or through the weld splash, they are in contact with heavy metals and minerals present in the wire, especially when the skin is hit by a weld splash because, due to its elevated temperature, the splash causes chemical burns. It was recommended to use sunscreen, especially during welding activity and when exposed to solar radiation and the use of welding gloves during activity and then proper hand washing in order to minimize contact with metals. 

Concerning the respiratory system, chemical risk factors were dealt with which apprentices are exposed to because they breathe the fumes resulting from the burning of metals during welding activity. The composition of the wire E71T-1 was again referred to as follows the question about the importance of a breathing mask with filter, a respiratory mask with filter being provided by the technical programme for training which protects against dust and fumes from welding. It is important to use, under the welding mask, a breathing mask with filter, because, without it, the welders will be inhaling dust and fumes from the welding process. Besides the chemical compounds present in fume welding, apprentices are also in contact with gases (acetylene and carbon dioxide) that are released during the opening of the flame. Unfortunately, the mask provided does not protect against inhalation of gases. For these reasons, it was recommended that apprentices do not remain in the environment of the welding practice rooms unnecessarily and/or without the protection of the respiratory mask with filter. Physical activities were recommended that promote breathing, such as races, in order to encourage gas exchange. 

For the auditory system approach, the physical risk factor was noise. The apprentices were informed about exposure to 89-90 dB from the welding machine during the practical activity. During the practical classes of each class, about 14 welding machines are used. The noise is caused by exhaust fans, which exceed the limit of 105 dB, which is the imposed limit for occupational exposure without proper protection. In addition, most apprentices use earplugs, such as headphones, which offer less protection than earplugs, unlike earmuffs. Apprentices were questioned on how they perform ear cleaning during the activities and practices of welding and on shared earplugs among apprentices. Some apprentices reported not performing ear cleaning and that they never lent earplugs. Daily cleaning with soap and water for earplugs recommended and advice was was reinforced of not lending earplugs because of the ease of transmission of bacteria by this route. 

For the musculoskeletal system, the following physiological risk factors were approached: performing repetitive movements, staying in the same posture for long periods, and sometimes incorrect posture, risk factors which apprentice welders are exposed to. To minimize exposure to these risk factors, the apprentices were asked to perform stretching exercises. During the exercises, apprentices were instructed to carry out the activity of stretching before and after welding practice and at intervals of 10 minutes, after 50 minutes of welding activity. In addition to these recommendations, after exposure of the systems, the following general recommendations were made: prioritize foods rich in iron and calcium to promote the excretion of manganese; prioritize foods rich in vitamin C to facilitate iron absorption; prioritize food rich in fibre to facilitate removal of manganese and other metals by feces, since only some of the manganese is eliminated in the urine.

### 3.6. Real Communication of Risk Perception and Occupational Accidents

**Figure 1:**
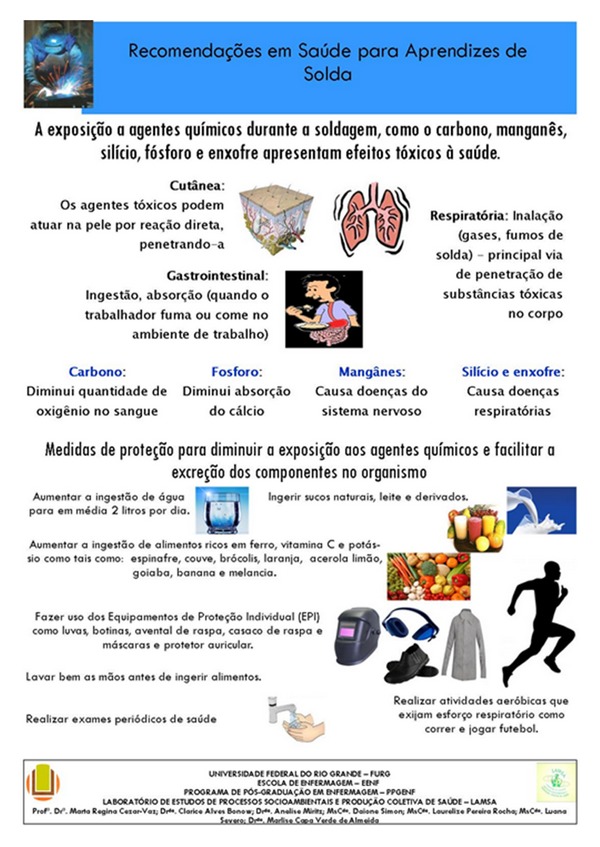
Poster provided from LAMSA for apprentice welders.

To continue the RC, the results of this research were presented. This approach focused on returning the perception of risk factors (physical, chemical, biological, and physiological) to which apprentices are exposed and occupational accidents arising from the characteristics of their activity. The presentation was concluded with the delivery of explanatory posters which were placed in the welding practice rooms, so that by looking at the poster, the implementation of protective measures during welding activity and minimization of exposure to risk factors would be stimulated (Figure 1).

## 4. Discussion

This study contributes to an understanding of the perception of risk factors and the occurrence of occupational accidents with apprentice welders. As regards the perception of risk factors that were identified, risks were reported in decreasing order: physical, chemical, physiological, and biological. Regarding accidents occurring to apprentices welders, it was found that the accidental skin burn was the most frequently mentioned (27.3%), and 26.1% of these accidents occur during apprenticeship activity. The apprentice welders are continually in contact with weld splash and hot metal objects, depending on the activity they perform, which can cause a greater number of such accidents [33]. It should also be considered that apprentices are in an apprenticeship process and knowledge essential to achieve consistently good welds is acquired not during theoretical activities but during practice [34]. However, opportunities to assess and improve the skills of “natural” security during practice are lower than in the workplace. Loss of control is rare, so individuals are only occasionally aware of the security requirements [35]. 

The findings also suggest that the perception of chemical risk and the occurrence of accidents involving this risk were more frequently present. This risk perception, related to the chemical risk and chemical occupational accident, is due to the raw material that the apprentices handle during the welding activity, for example, the hot metal [31]. The metals which apprentice welders are in contact with include aluminum [36], stainless steel [37], cadmium [27], chromo [38], lead [39], copper [28], manganese [28], molybdenum [28], and nickel [39]. These chemicals may generate hazardous fumes during welding activity. According to the International Labour Organization [31], these metals are related to risk factors and the occurrence of chemical accidents, when the welders are hit by weld splash or hot metal particles and because of exposure to metal fumes.

Among the chemical risks, 75.8% of apprentice welders recognized the gases with which they deal during welding activity as risk factors, 56.5% identified the dust present in the apprenticeship environment, and 36% the fumes from welding. Study indicates that the welding fumes from the chemical compound, stainless steel, can cause acute lung injury and the size of the inhaled particles and exposure time are significant factors in the welding, which must be considered in the development of protective strategies [37]. Lung function and respiratory symptoms in welders were therefore investigated in a case-control study [40], noting significantly higher prevalence of respiratory symptoms (dyspnea and secretion) in welders. The study suggests that the welders are at risk of developing respiratory symptoms and decreased lung function, although the concentrations of metal fume were lower than the recommended limit by the American Conference of Industrial Hygienists (ACGIH).

Another important pathology in welders is lung cancer. Cohort [26] conducted with male welders, from 1964 to 1984, showed that the incidence rate of lung cancer was higher. An important chemical compound, carcinogen, found in welding activity is chromo. Studies suggest that chronic occupational exposure during welding activity can raise levels of damage to genetic material and inhibit the repair of the same [38, 41]. However, a study to identify occupational exposures associated with increased incidence of breast cancer in men found that welders are not considered a risk group [42].

Another exposure assessment for lead, chromo, and nickel in welding work and the relation with chromosomal damage, evaluated 60 welders: men, divided into two groups, group 1, working without PPD and group 2, who work with PPD. The metal concentration was analyzed in the blood and urine of the workers. The analysis showed that workers in group 1 had a higher frequency of chromosomal damage than group 2 [39]. 

The association of exposure of welders and operators to lead, cadmium, and manganese and nervous system damage, found that exposure of welders is greater than operators. There were significant differences in the relationship between damage to the nervous system and exposure to lead and manganese [43]. Cadmium exposure in welders was analyzed by linking such exposure to renal dysfunction [27]. Cadmium has also been investigated in combination with noise [44], indicating probable ototoxic metal when associated with noise exposure. In Brazil, regulating Norm number 15 considers that welding using compound cadmium is an unhealthy operation of maximum degree [45].

A longitudinal study of apprentice welders showed a significant association between welding-related metal fume and respiratory symptom fever as well as a decrease in lung function values after 15 months in welding school [17, 46].

This study documented and reported that occupational chemical accidents are higher in apprentice welders who have greater experience. The most common chemical accident between apprentice welders was skin burns. Occupational burns are divided into three categories. Thermal burns include events that result from high levels of heat caused by explosions, flame, radiant-heat, and direct contact with hot surfaces. Electric injuries result from electrical explosions, flashes, or direct contact with an electrical current. Chemical burns result from the reaction of biologic tissues with chemical materials [47]. 

Specifically with apprentice welders, burns that can occur include thermal burns and chemical burns. There is a study which describes the occurrence of work-related injuries from thermal, electrical, and chemical burns among electric utility workers, among these, the welders. Welders (not a common occupation in the electric utility workforce) had the highest age-adjusted injury rates for all burn-related injuries (61.57 per 10,000 employee-years) and for thermal/heat burns (40.87 per 10,000 employee-years). It is understood that in the case of welding activity, a thermal burn may constitute a chemical burn, as contact with the chemical compounds present in the metal that cause thermal burns can cause a chemical burn [33]. 

**Figure 2:**
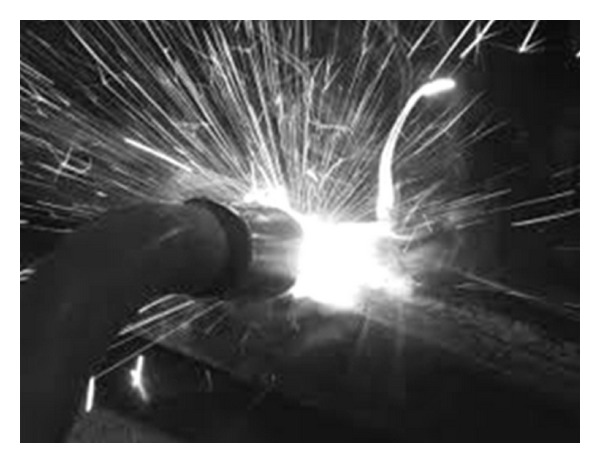
Opening for electric arc welding activity.

Another risk self-reported by the apprentice welders is physical, mentioned by 96.9% of the apprentices, the main physical risk being heat (79.5%). The self-reporting of heat by apprentice welders arises from the non-ionizing radiation produced by welding activity. More specifically, heat is produced during the opening of the electric arc (Figure 2), which consists of an electric discharge.

Study findings show that the intensity and wavelength of nonionizing radiation produced would depend on many factors, such as the type of welding process, welding parameters, the composition of metals, fluxes, and any coatings that may be on the base material. Moreover, the radiation exposure time was considered combinable with each 8 hour exposure within a 24-hour period. Therefore, two exposures of 5 minutes during a workday can be considered as a single 10-minute exposure. The research results show that the minimum safe distance for 1 minute is 32 cm [48].

Another study [49] conducted to quantify the risk of arc eye during welding activity showed that the maximum exposure without protection is around 0.47 to 4.36 seconds. For this reason, it is important that welders avoid direct exposure to light to initiate the welding arc. Moreover, they must use personal protective equipment appropriate for the eyes and for the type of weld. 

The apprentices also identified the physical risk factor during welding activity. Study [24] sought to examine the prevalence of cases suggestive of noise-induced hearing loss in welders. Although the number of workers who have experienced the disease was low, the study was able to determine some risk factors within and outside the workplace, such as regular use of hearing protection and exposure to extra-occupational noise sources. Moreover, excessive noise in the workplace can be a risk factor for the development of vocal disorders [50].

The physiological risk was reported by 86.3% of apprentice welders, showing mainly poor posture and repetitive stress. These factors are exacerbated by excessive vibration during welding activity. Research [51] performed with different workers showed that, specifically for welders, vibration may be associated with back pain. 

Study [25] sought to identify symptoms of musculoskeletal disorders in metallurgical workers, analyzing the administrative sector and the sector of production/operations. The authors concluded that the prevalence of these symptoms is high and suggested risk factors such as age (from 33 years), low education level, and occupation (workers production/operational sector/showed more symptoms with manual labor performance). 

The biological risk was identified by 51.5% of the apprentices, especially as major contaminating microorganism contaminations were bacteria and fungi. Regarding biological risk perception, one study [52] identified the workers' perception of imminent infectious disease risk. The results showed that risk perception varied as a function of the frequency of the workers' exposure to contaminated fluids, knowledge of customers' diseases, and history of previous accidents [19].

From the presented exploratory study, there was a socio-environmental education intervention (SEI) for apprentice welders. The SEI during the apprenticeship process encourages apprentices to think about the risk factors in the workplace that can cause illness or accidents. In the specific case of the SEI described in this study, means were used to encourage apprentices to visualize ways of minimizing risk factors and, therefore, occupational accidents, as is the case of PPD, which can be used in the workplace and also visualization of strategies for minimization of risk factors. 

It is believed that RC, through a process of education, can modify individual behavior because it is a process in which apprentices perceive and multiply knowledge in their work/apprenticeship environment and thus interfere with collective work conditions.

## 5. Conclusions

In conclusion, apprentice welders realize that they are exposed to risk factors, especially chemical risk factors, due to their workplace being particularly dangerous. The frequency of occupational accidents during apprenticeship leads to a state that allows the perception of risk factors of the accident to be realized by the apprentice.

Such evidence confirms the findings of the literature on risk factors that apprentice welders face during activity and in similar situations to those found in this particular study. It is understood that the perception of apprentices regarding a particular set of occupational risks is essential to be able to develop an effective RC as a positive tool for teaching and learning. Study limitations as, for overall risk management to become a reality, it is necessary to conduct studies that accompany apprentices in the workplace and develop comparisons and intervention necessary to promote the health and safety of them. 

Thus, one of the theoretical and operational propositions of HPDWEP is to improve the evidence of research and socio-environmental education intervention for perception and for RC to be used as a tool for teaching and learning for the promotion of socio-environmental health of workers in their working environment.

## Figures and Tables

**Table 1:** Demographic characteristics of the study subjects (*n* = 161)*.

Variables	Categories	*n*	Percent (%)
Gender	Male	132	82.0
	Female	27	16.8

Marital status	Single	87	54.0
	Married	64	39.8
	Separated	5	3.1
	Widowed	1	0.6

Skin colour/ethnic origen	White	95	59.0
	Black	31	19.3
	Brown	25	15.5
	Asian	3	1.9
	Indigenous	3	1.9

Schooling	Elementary school, incomplete	15	9.3
	Elementary school	8	5.0
	Secondary school, incomplete	30	18.6
	Secondary school	84	52.2
	Higher education, incomplete	11	6.8
	Higher education	8	5.0
	Postgraduate education, incomplete	2	1.2

*Numbers for each item may total less than total numbers because of missing values.

**Table 2:** Perception of apprentice welders about physical, chemical, biological, and physiological risk factors (*n* = 161).

Risk factors	*n*	Percent (%)
Physical		
Heat	128	79.5
Noise	102	63.4
Ionizing radiation	83	51.6
Nonionizing radiation	41	25.5
Vibrations	24	14.9
Abnormal pressures	18	11.2
Moisture	13	8.1
Cold	9	5.6
Chemical		
Gases	122	75.8
Dust	91	56.5
Chemical products	58	36
Fumes	58	36
Vapours	51	31.7
Mist	15	9.3
Fog	8	5
Biological		
Bacteria	43	26.7
Fungi	38	23.6
Virus	28	17.4
Bacilli	8	5
Parasites	6	3.7
Protozoa	6	3.7
Physiological		
Poor posture	96	59.6
Repetitive strain	56	34.8
Inadequate ventilation	53	32.9
Use of inappropriate equipment	47	29.2
Inadequate lighting	35	21.7
Rhythm of overwork	32	19.9
Requirement productivity	30	18.6
Machines and/or inadequate equipment	29	18

**Table 3:** Occupational accidents reported by apprentice welders (*n* = 161).

Occupational accidents	*n*	Percent (%)
Skin burns	44	27.3
Electric shock	8	5.0
Eye irritation	20	12.4
Explosion caused by flammable gases	4	2.5
Explosion caused by inadequate electrical installations	3	1.9
Cutting of the hands	3	1.8
Injury caused by falling pieces	1	0.6
Fall from scaffolding	1	0.6
Eye burn	2	1.2

## References

[1] Sabitu K, Iliyasu Z, Dauda MM (2009). Awareness of occupational hazards and utilization of safety measures among welders in Kaduna metropolis, Northern Nigeria. *Annals of African Medicine*.

[2] Jayawardana P, Abeysena C (2009). Respiratory health of welders in a container yard, Sri Lanka. *Occupational Medicine*.

[3] Rolland P, Gramond C, Lacourt A (2010). Occupations and industries in France at high risk for pleural mesothelioma: a population-based case-control study (1998–2002). *American Journal of Industrial Medicine*.

[4] Ibfelt E, Bonde JP, Hansen J (2010). Exposure to metal welding fume particles and risk for cardiovascular disease in Denmark: a prospective cohort study. *Occupational and Environmental Medicine*.

[5] Sardas S, Omurtag GZ, Tozan A, Gül H, Beyoglu D (2010). Evaluation of DNA damage in construction-site workers occupationally exposed to welding fumes and solvent-based paints in Turkey. *Toxicology and Industrial Health*.

[6] Simon DP, Gutierrez LLP, Macedo SMD (2009). Hematologic and morphocytologic alterations in biologic fluids of workers of the industrial district of Erechim, RS. *Revista Brasileira de Análises Clínicas*.

[7] Slovic P (2000). *The Perception of Risk*.

[8] Pidgeon N (1992). *Risk Perception. Risk Analysis, Perception and Management*.

[9] Douglas M, Wildavsky A (1982). *Risk and Culture. An Essay on the Selection of Technological and Environmental Dangers*.

[10] Slovic P (1987). Perception of risk. *Science*.

[11] Hayes MV (1992). On the epistemology of risk: language, logic and social science. *Social Science and Medicine*.

[12] Barnett J, Breakwell GM (2001). Risk perception and experience: hazard personality profiles and individual differences. *Risk Analysis*.

[13] Eden D, Ravid G (1982). Pygmalion versus self-expectancy: effects of instructor- and self-expectancy on trainee performance. *Organizational Behavior and Human Performance*.

[14] Tassava CJ (2003). Weak seams: controversy over welding theory and practice in American shipyards, 1938–1946. *History and Technology*.

[15] Benway EA (2010). As national welder shortage looms, proper training becomes a critical asset. *Plant Engineering*.

[16] Bloom AD, Sewell G, Neriishi S (1980). Chromosomal abnormalities among welder trainees. *Environment International*.

[17] El-Zein M, Malo J-L, Infante-Rivard C (2003). Incidence of probable occupational asthma and changes in airway caliber and responsiveness in apprentice welders. *European Respiratory Journal*.

[18] Laohaudomchok W, Lin X, Herrick RF (2011). Neuropsychological effects of low-level manganese exposure in welders. *NeuroToxicology*.

[19] Sjöberg L (2000). The methodology of risk perception research. *Quality and Quantite*.

[20] Leoni T (2010). What drives the perception of health and safety risks in the workplace? Evidence from European labour markets. *Empirica*.

[21] Public Health Service US (1995). Risk communication: working with individuals and communities to weigh the odds. *Prevention Report*.

[22] (1999). *Occupational Safety and Health Act of 16 June 1999*.

[23] Organização Pan-Americana da Saúde no Brasil (2001). *Doenças Relacionadas ao Trabalho: Manual de Procedimentos Para os serviços de saúde*.

[24] Guerra MR, Lourenço PMC, Bustamante-Teixeira MT (2005). Prevalence of noise-induced hearing loss in metallurgical company. *Revista de Saúde Pública*.

[25] Picoloto D, da Silveira E (2008). Prevalence of musculoskeletal symptoms and associated factors among metal industry workers in Canoas—RS. *Ciência & Saúde Coletiva*.

[26] Sørensen AR, Thulstrup AM, Hansen J (2007). Risk of lung cancer according to mild steel and stainless steel welding. *Scandinavian Journal of Work, Environment and Health*.

[27] Ding X, Zhang Q, Wei H, Zhang Z (2011). Cadmium-induced renal tubular dysfunction in a group of welders. *Occupational Medicine*.

[28] Balkhyour MA, Goknil MK (2010). Total fume and metal concentrations during welding in selected factories in Jeddah, Saudi Arabia. *International Journal of Environmental Research and Public Health*.

[29] Cezar-Vaz MR (2010). *Risks and Occupational Diseases: An Integrated Study in Different Work Environments*.

[30] American Welding Society (1991). *Handbook: Welding Processes*.

[31] (2000). *Welder, Arc: International Hazard Datasheets on Occupation*.

[32] National Library of Medicine Cataloging in Publication (2002). *Communicating in a Crisis: Risk Communication Guidelines for Public Officials*.

[33] Fordyce TA, Kelsh M, Lu ET, Sahl JD, Yager JW (2007). Thermal burn and electrical injuries among electric utility workers, 1995–2004. *Burns*.

[34] Evans GT, Butler J (1992). Expert models and feedback processes in developing competence in industrial trade areas. *Australian Journal of TAFE Research and Development*.

[35] Middleton H, Pavlova M, Roebuck D (2002). *Learning in Technology Education Challendges for the 21st Century*.

[36] Kiesswetter E, Schäper M, Buchta M (2009). Longitudinal study on potential neurotoxic effects of aluminium: II. Assessment of exposure and neurobehavioral performance of Al welders in the automobile industry over 4 years. *International Archives of Occupational and Environmental Health*.

[37] Leonard SS, Chen BT, Stone SG (2010). Comparison of stainless and mild steel welding fumes in generation of reactive oxygen species. *Particle and Fibre Toxicology*.

[38] Sudha S, Kripa SK, Shibily P (2011). Biomonitoring of genotoxic effects among shielded manual metal arc welders. *Asian Pacific Journal of Cancer Prevention*.

[39] Iarmarcovai G, Sari-Minodier I, Orsière T (2006). A combined analysis of XRCC1, XRCC3, GSTM1 and GSTT1 polymorphisms and centromere content of micronuclei in welders. *Mutagenesis*.

[40] Loukzadeh Z, Sharifian SA, Aminian O, Shojaoddiny-Ardekani A (2009). Pulmonary effects of spot welding in automobile assembly. *Occupational Medicine*.

[41] Danadevi K, Rozati R, Banu BS, Grover P (2004). Genotoxic evaluation of welders occupationally exposed to chromium and nickel using the Comet and micronucleus assays. *Mutagenesis*.

[42] Villeneuve S, Cyr D, Lynge E (2010). Occupation and occupational exposure to endocrine disrupting chemicals in male breast cancer: a case-control study in Europe. *Occupational and Environmental Medicine*.

[43] Wang XL, Yang YJ, Wang X, Xu SQ (2006). The effect of occupational exposure to metals on the nervous system function in welders. *Journal of Occupational Health*.

[44] Abreu MT, Suzuki FA (2002). Audiometric evaluation of noise and cadmium occupationally exposed workers. *Revista Brasileira de Otorrinolaringologia*.

[45] Ministério do Trabalho e do Emprego (2008). *Legislação de Segurança e Medicina do Trabalho*.

[46] El-Zein M, Infante-Rivard C, Malo JL, Gautrin D (2005). Is metal fume fever a determinant of welding related respiratory symptoms and/or increased bronchial responsiveness? A longitudinal study. *Occupational and Environmental Medicine*.

[47] Casini V (1998). Overview of electrical hazards. *Worker Deaths by Electrocution: A Summary of surveillance Findings and Investigative Case Reports*.

[48] Lyon T (2002). Knowing the dangers of actinic ultraviolet emissions. *Welding Journal*.

[49] Okuno T, Ojima J, Saito H (2010). Blue-light hazard from CO_2_ arc welding of mild steel. *The Annals of Occupational Hygiene*.

[50] Ubrig-Zancanella MT, Behlau M (2010). Relation between work environment and voice deviation in metallurgic workers. *Revista da Sociedade Brasileira de Fonoaudiologia*.

[51] Chen Y, McDonald JC, Cherry NM (2006). Incidence and suspected cause of work-related musculoskeletal disorders, United Kingdom, 1996–2001. *Occupational Medicine*.

[52] Jovic-Vranes A, Jankovic S, Vukovic D, Vranes B, Miljus D (2006). Risk perception and attitudes towards HIV in Serbian health care workers. *Occupational Medicine*.

